# Amino Acid-Based Formula vs. Extensively Hydrolyzed Formula in the Treatment of Feeding Intolerance in Preterm Infants: Study Protocol for a Randomized Controlled Trial

**DOI:** 10.3389/fnut.2022.854121

**Published:** 2022-05-30

**Authors:** Qin Zhong, Qi Lu, Nan Peng, Xiao-Hua Liang

**Affiliations:** ^1^Department of Neonatology, Children's Hospital of Chongqing Medical University, National Clinical Research Center for Child Health and Disorders, Ministry of Education Key Laboratory of Child Development and Disorders, Chongqing Key Laboratory of Pediatrics, Chongqing, China; ^2^Department of Clinical Epidemiology and Bioinformatics, Children's Hospital of Chongqing Medical University, Chongqing, China

**Keywords:** feeding intolerance, preterm infant, amimo acid-based formula, extensively hydrolyzed formula, randomized controlled trial, protocol

## Abstract

**Background:**

Feeding intolerance is a common problem in preterm infants, which is associated with an increased risk of infections, prolonged hospitalization, and increased economic costs. When human milk is not available, formula feeding is required. Amino acid-based formula and extensively hydrolyzed formula could be considered for use for severe feeding intolerance. A recent Cochrane meta-analysis found that preterm infants fed extensively hydrolyzed formula compared with standard formula could not reduce the risk of feeding intolerance and necrotizing enterocolitis, and weight gain was slower. Some studies reported that preterm infants fed amino acid-based formula could reduce the gastric residual volume. We hypothesize that amino acid-based formula can improve feeding intolerance and establish full enteral feeding more rapidly in preterm infants compared with extensively hydrolyzed formula.

**Method:**

The randomized, prospective, controlled trial was conducted at the Children's Hospital of Chongqing Medical University (Chongqing, China). A total of 190 preterm infants with gestational age <32 weeks or birth weight <1,500 g and with a diagnosis of feeding intolerance were included. Patients were randomized to an amino acid-based formula-fed group and an extensively hydrolyzed formula-fed group. The primary outcome is the time (days) to reach full enteral feedings. Secondary outcomes include duration of vomiting and abdominal distension, gastric residual volume, body weight, length and head circumference during hospitalization, length of hospital stay (days), cost of hospitalization, time (days) of parenteral nutrition, change of abdomen circumference, main serum parameters, and incidence of adverse events.

**Discussion:**

The successful implementation of our study will provide robust evidence for formula alternatives in preterm infants with feeding intolerance.

**Clinical Trial Registration:**

www.ClinicalTrials.gov, identifier: NCT05347706.

## Background

Globally, approximately 15 million premature infants are born every year, with this number increasing progressively (1). Due to the immature digestive, absorptive, and immunologic functions, preterm infants are particularly susceptible to mucosal inflammation and bacterial overgrowth, which can lead to feeding intolerance (FI) (2). FI frequently occurs in premature infants, especially in those with a gestational age of <32 weeks or a birth weight of <1,500 g (3, 4). FI is defined as the inability to digest enteral feeding and is characterized by increased gastric residuals, abdominal distension, vomiting, or both (5). This delays the establishment of full enteral nutrition and extends the duration of parenteral nutrition, thus increasing the risk of infections, prolonging the length of hospital stay, and increasing economic costs (6).

At present, there are some prevention and treatment measures for FI including optimization of enteral nutrition, modification of feeding methods, use of probiotics or medicine, and nursing interventions, but these measures are not fully effective (7, 8). The feeding strategy for FI is an important clinical challenge for neonatologists. Associated with less feeding intolerance, human milk (HM) is recommended by the World Health Organization (WHO) as the first-choice milk for preterm. However, HM is not always available because of the lack of breast milk banks, diseases of the mother, and geographic factors, and therefore, formula feeding is required.

Currently, the alternative formulas include Preterm Formula (PF), Partially Hydrolyzed Formula (PHF), Extensively Hydrolyzed Formula (EHF), Amino Acid-Based Formula (AAF), etc. PF is used in preterm infants when human milk is not available (9). Clinically, PHF, EHF, and AAF are commonly used in treatment for moderate to severe cow's milk protein allergy and prevention for patients at high risk for allergy (10, 11). PF containing intact protein may not be appropriate for infants with FI (12, 13). Recently, a guideline mentioned that PHF, EHF, and AAF could be considered for use in severe feeding intolerance (8).

Mihatsch et al. (14) reported that EHF improved the feeding tolerance and enabled a more rapid establishment of full enteral feeding compared with standard PF in preterm infants. The use of EHF could reduce acid gastro-esophageal reflux in preterm infants with FI (13). A recent Cochrane meta-analysis found that existing data did not support conclusions that feeding PHF or EHF affected the risk of FI or necrotizing enterocolitis (NEC), but the data that could be abstracted from published studies for analysis were limited (3). Raimondi et al. (15) reported that preterm infants with severe feeding intolerance significantly and rapidly reduced the gastric residual volume after AAF introduction. Jang et al. (16) found the fecal calprotectin levels in AAF-fed infants with FI were significantly lower than those in the HM- or PF-fed infants with FI and showed improvement in the symptoms and signs of FI. However, these studies have some defects, such as small sample sizes and no randomization. Which formula is more suitable for preterm infants with FI? No scientific evidence is available at present.

Based on previous research, we design a randomized, prospective, clinical trial of AAF vs. EHF in feeding intolerance. We hypothesize that AAF can improve feeding intolerance and establish full enteral feeding more rapidly compared with EHF.

### Aim

The aim of the present study is to investigate whether AAF enables a more rapid establishment of full enteral feeding in preterm infants with FI compared with EHF and help establish improved guidelines and feeding practices.

## Method/Design

### Study Design

**Figure 1:**
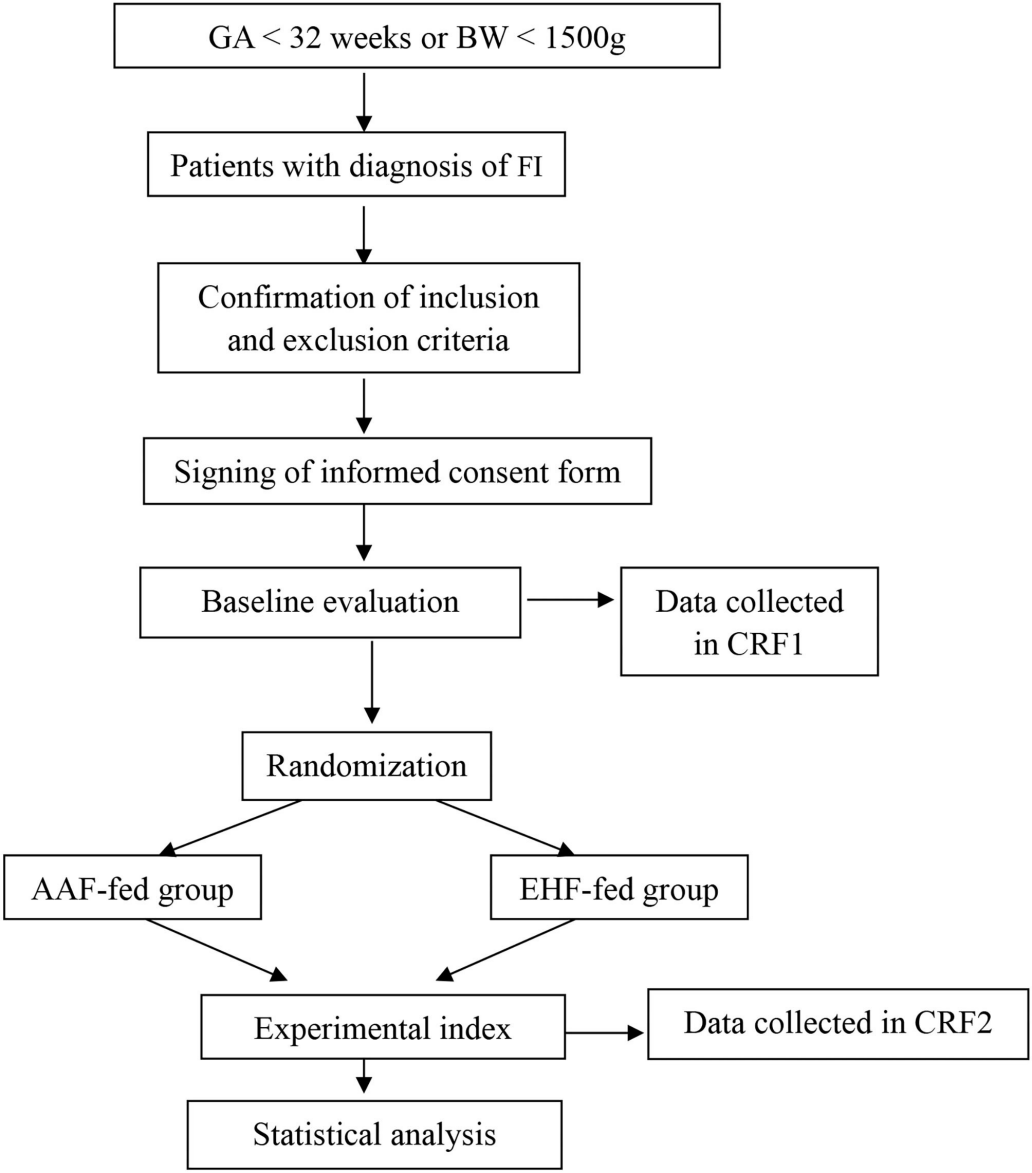
The overall design of the study.

This study is designed as a single*-*center, randomized, prospective, blinded clinical trial. A total of 190 preterm infants with gestational age <32 weeks or birth weight <1,500 g with a diagnosis of FI were included in the study. They were randomized to the AAF group or the EHF group. Figure 1 shows the overall design of the study. Table 1 shows the data collection schedule.

**Table 1:** Data collection schedule.

	**Staff member**	**T1**	**T2**	**T3**	**T4**	**T5**
Symptom of FI	Neonatal physician	**×**				
Informed consent	Research assistant	**×**				
Contact information	Research assistant	**×**				
Sex	Neonatal physician		**×**			
Gestational age	Neonatal physician		**×**			
Mode of delivery	Neonatal physician		**×**			
Apgar score	Neonatal physician		**×**			
Maternal issues	Neonatal physician		**×**			
Weight	Neonatal nurse		**×**			**×**
Gastric residual volume	Neonatal nurse			**×**	**×**	
Abdominal circumference	Neonatal nurse			**×**	**×**	
Length	Neonatal nurse		**×**			**×**
Head circumference	Neonatal nurse		**×**			**×**
Time to reach total enteral nutrition	Neonatal physician					**×**
Duration of abdominal distension	Neonatal physician					**×**
Duration of vomiting	Neonatal physician					**×**
Length of hospital stay	Neonatal physician					**×**
Cost of hospitalization						**×**
Time of parenteral nutrition	Neonatal physician					**×**
Main serum parameters	Neonatal physician			**×**	**×**	
Incidence of adverse events	Neonatal physician					**×**

*T1 screening; T2 baseline evaluation; T3 beginning of intervention; T4 end of intervention; T5 discharge*.

### Study Setting

All subjects were recruited in the Children's Hospital of Chongqing Medical University (Chongqing, China), a tertiary university hospital that has a 250-bed neonatal unit with an annual admission rate of around 8,000 neonates in the past 2 years.

### Study Population

Table 2 specifies all inclusion and exclusion criteria.

**Table 2:** Inclusion and exclusion criteria.

Inclusion criteria
• Admission between December 2021 and December 2023.
• Gestational age (GA) <32 weeks or birth weight (BW) <1,500 g, appropriate for gestational age, admitted to Department of neonatology, Children's Hospital of Chongqing Medical University within the first 24 h after birth, maximal enteral intake <50 ml/kg/day
• Patients are fed with PF when HM is not available after admission
• Meet the diagnostic criteria of FI. Currently, a clear and universal definition of FI is lacking, FI is defined as follows with reference to relevant literature (5, 16–18): One or two of the criteria below are met: (1) gastric residual volume ≥50% of the previous feeding volume (≥ two times within 24 h), with the presentation of vomiting and/or abdominal distension; (2) feeding plans fail: including feeding withheld or decrease > 6 h, or not increased >24 h (5)
• Parental consent has been obtained
Exclusion criteria
• Perinatal asphyxia: (1) Apgar score of <4 at 5 min; (2) Fetal umbilical artery acidemia: pH <7.00 and/or base deficit worse than or equal to minus 12 mmol/L; (3) A significant peripartum or intrapartum hypoxic-ischemic event (e.g., uterine rupture, placental abruption, cord prolapse, amniotic fluid embolism, fetal exsanguination from a vasa previa or massive feto-maternal hemorrhage, etc.).
• Potential metabolic or chronic disease, congenital abnormality, or any other diseases that may affect feeding ability, normal growth, and development before recruitment
• Patients who need surgical treatment under general anesthesia (ligation of patent ductus arteriosus is excluded) before or on the date of randomization
• Blood pressure is unstable (allowing for dopamine <5 μg/kg/min)
• Ventilator dependence or FiO2 >40% on the date of randomization (allowing for nasal intubation, CPAP, and/ or oxygen mask)
• Grade III or IV intraventricular hemorrhage is diagnosed before or on the date of randomization

#### Termination Criteria

Discharge in a stable condition with advice.

#### Exit Criteria

1. Death or discharge before total enteral nutrition.
2. Guardian request to withdraw from the study.
3. Sepsis, NEC, use of ventilatory support, or critical illness is not suitable for continuing to participate in the study.

### Sample Size

This is a prospective randomized controlled study. Patients meeting the inclusion and exclusion criteria are randomly divided into two groups, group A (AAF) and group B (EHF). Time to reach full enteral feeding is the primary outcome measure; the same research is unreported at present. According to Raimondi's study (15) and experience in clinical practice, the mean time to full enteral feeding in group A is 23.6 ± 15.6 days, accounting for a 30% disparity, the time to full enteral feeding between the two groups differs by approximately 7.08 days. Hypothesis tests are two-sided with a significance level of 5%, while the statistical power is set at 80%. In this study, Zα = 1.96, Zβ = 0.84, σ represents standard deviation, δ represents the difference between the mean values in the two groups, and the formula for calculating sample size is as follows:


$$\begin{array}{l} n=\frac{{\left(Z_{\alpha }+Z_{\beta }\right)}^{2}*2\sigma^{2}}{\delta^{2}} \end{array}$$

After calculation, a sample size of 76 per group is required, accounting for a 20% miss rate. Ninety-five patients per group need to be included, and the planned sample size of this study is at least 190 patients in total.

### Randomization

Randomization will be performed using the SPSS software package (version 22.0) using a random number generator in a 1:1 ratio. A statistician who is not involved in recruitment and subsequent data analysis will generate the randomization list, and the list will be concealed. After random allocation lists have been generated, the allocation group (AAF or EHF) will be stored in sequentially numbered, sealed, opaque envelopes. These envelopes will be opened by a third-party individual at each study enrolment after baseline measures have been obtained.

The study will be double*-*blinded. A nutritionist who is not directly involved in inpatient care will prepare and label the formula with the participant's randomization number, date, and time of feeding, and then deliver the formula to the nurse caring for the infant. The medical and nursing teams caring for the infants will be unaware of the type of formula used for feeding. The trial statistician will perform data analysis and be kept unaware of treatment group assignment until the results are finalized.

### Feeding Schedule

**Figure 2:**
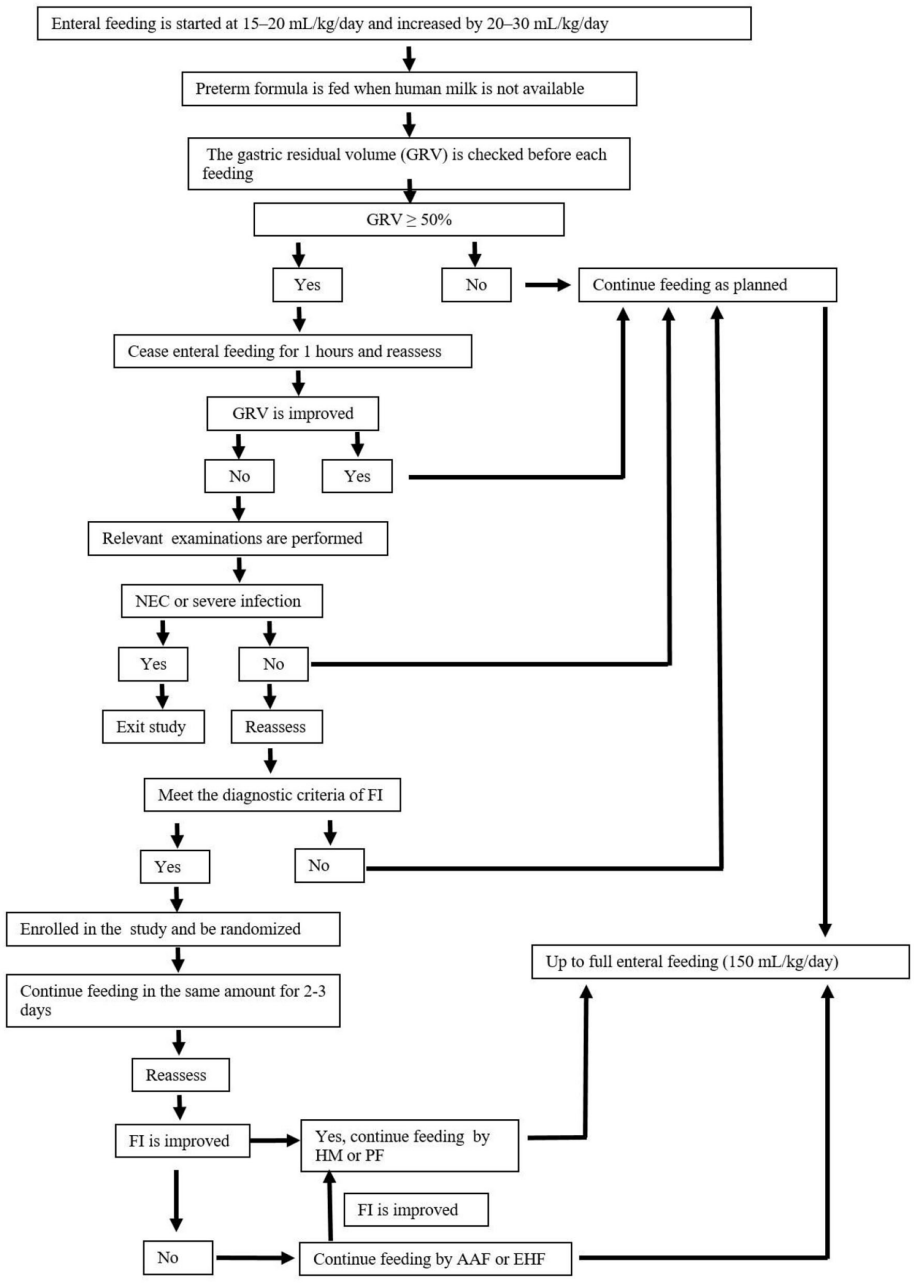
Feeding schedule.

According to the guideline for nutrition support (19), enteral feeding is started within the first 24 h of life. Human milk is encouraged, and preterm formula is fed when human milk is not available due to the mother's or family's condition. Patients with FI are temporarily fed with AAF (Neocate® Nutricia, London, UK) or EHF (Alfare® Nestle, Netherlands) instead of HM or PF. Nutritional feeds start at 15–20 mL/kg/day and are increased by 20–30 mL/kg/day. The gastric residual volume (GRV) is checked before each feeding. If GRV is ≥ 50% of the previous feeding, then enteral feeding was ceased for 1 h, and patients are reassessed. If GRV is not improved, complete blood count, C-reactive protein (CRP), and abdominal X-rays are performed and assessed. If these results can rule out NEC and severe infection, patients are fed in the same amounts for 2–3 days. Enteral feeding is increased at the same rate until full enteral feeding (150 mL/kg/day) every 3 h is reached. Enteral feedings are withheld: in the presence of bloody or biliary gastric residuals, in cases of abnormal abdominal examination, and/or in cases of abnormal abdominal X-ray. As soon as the residuals, the abdominal examination, and/or X-ray return to normal, feeding is resumed with AAF or EHF at the same speed. Once the patients with FI show clinical improvement, AAF or EHF feeding is discontinued, and the previous feeding, either HM or PF is resumed. When full enteral nutrition is insufficient, all patients receive parenteral nutrition solutions (6), which are gradually decreased with increasing enteral feeding. Other routine treatments for FI are given in the two groups according to the guidelines. Table 3 shows the study formulas. Figure 2 shows the feeding schedule.

**Table 3:** Study formulas.

**Characteristics**	**Study formula**	
	**AAF**	**EHF**
Nutrient density (KJ/100ml)	279	284
Nutrient composition per 100 ml		
Total Protein (g, % calories)	2.0,12%	1.82,11%
Total Carbohydrate (g, % calories)	7.0,42%	7.5,43%
Total Fat (g, % calories)	3.4,46%	3.39,46%
Arachidonic acid (ARA), mg	11.3	8.5
Docosahexaenoic acid (DHA), mg	11.3	8.5
Minerals		
Phosphate (mg/100 ml)	59	46
Calcium (mg/100 ml)	85	68
Iron (mg/100 ml)	1.1	0.7
Osmolarity (mOsm/L)	310	170

### Variables Studied

The investigator who is responsible for recruitment will perform all the evaluations and remain blinded to the status of the patients with regard to the intervention throughout the study.

Table 4 shows the demographic variables. The following variables: age, sex, birth weight, birth length, delivery mode, gestational age, multiple gestations, family history, birth history, and history of present condition.

**Table 4:** Demographic variables.

**Variables**	**values**
Age Sex Gestational age Birth weight Delivery mode Multiple gestations Apgar score (1', 5', 10') Maternal health during pregnancy Adverse events at birth	Days Male/Female Weeks g vaginal delivery or cesarean section Yes/no 1-10

### Outcome

#### Primary Outcome

Time (days) to reach full enteral feedings, defined as a daily intake of ≥ 150 mL/Kg/day for 3 days in a row (age at the first day of achieving full enteral feedings as the indicator).

#### Secondary Outcome

1. Duration of vomiting and abdominal distension (a 24-h abdominal circumference increase of ≥1.5 cm).
2. Gastric residual volume (measured by aspirating with a 5-ml syringe before each feeding).
3. Body weight, length, and head circumference during hospitalization.
4. Length of hospital stay (days).
5. Cost of hospitalization.
6. Time (days) of parenteral nutrition.
7. Change of abdomen circumference (measured at a specific time every day).
8. Main serum parameters (blood, liver and kidney function, and electrolytes).
9. Incidence of adverse events.

**Study endpoint**: The day of discharge

**Follow*-*up plan**: No.

### Statistical Analyses

The statistical analysis is performed using the SPSS software package, version 22.0. The measurement data obeying the normal distribution are expressed as x ± s, and the *t-*test is used for comparison between groups. Non-normally distributed continuous variables are expressed as median with interquartile range and compared using the Mann-Whitney U test. The enumeration data are expressed by rate (%) and analyzed by the chi-square test or Fisher's exact test. *P* <0.05 is considered a significant statistical difference.

### Recruitment

First, persons in charge of recruitment and quality control are identified. Patients who met all inclusion criteria and none of the exclusion criteria are recruited in this study through the electronic medical record system. Then, their legal guardians are informed of the content of this study, written informed consent is signed, and a registration form is filled. The quality controller randomly chooses from guardians of patients over the course of recruitment and asks if they have read and understood the written informed consent form, to ensure that the rights of patients and guardians are safeguarded.

### Data Collection and Management

Data management and monitoring will be performed by using the ResMan Research Manager (http://www.medresman.org), and all data sets will be password protected. Only the investigators directly involved with the study will have access to the account number and password. Investigators correctly, completely, clearly, and timely record data in the case report forms (CRFs) according to original observation. The CRFs after inspection need to be transmitted to the data administrator of the clinical research in time. Data will be entered into the ResMan by the authorized researchers. Data entry will be by double entry, and matching will be conducted after inconsistent data has been reviewed. Original CRFs are stored in numerical order and kept in locked cabinets after the completion of data entry and review.

## Discussion

This randomized, prospective, clinical trial aims to assess the effects of AAF or EHF on improving FI. A total of 190 preterm infants with FI are included and randomly divided into two groups: the AAF-fed and EHF-fed groups. Time to reach full enteral feedings, duration of vomiting, abdominal distension and gastric residual volume, and other indicators are evaluated.

The mechanisms by which AAF improves feeding intolerance remain incompletely unclear. AAF is composed of free amino acids, no lactose, and a higher percentage of medium chain triglycerides. The amino acids are essential for intestinal function and have an important role in mucosal blood perfusion, growth, and immunity (20). AAF may promote gastric emptying, decrease the duration of parenteral nutrition, and reduce the risk of nosocomial infections and parenteral nutrition-associated complications.

However, there have been some concerns as to whether AAF and EHF meet the nutritional needs of preterm infants (16, 21). In this study, the patients with FI are fed AAF or EHF for a short term; once the clinical presentation of FI improved, HM or PF are switched back. Jang et al. (16) reported that the patients with FI who fed AAF for a short term showed improvement in the symptoms and signs of FI with proper body weight gain, and did not develop any adverse clinical signs or symptoms during AAF feeding. There was not any disadvantage in preterm infants on growth between the EHF-fed group and the PF-fed group in the long-term (12 weeks) study (22), and long-term treatment with AAF was safe and allowed adequate body growth (23).

The study also has some limitations that should be noted. Due to the imperfect follow-up system, the lack of post-intervention follow*-*up makes it difficult to judge long*-*term prognosis. Additionally, the study population are low birth weight preterm infants with a high risk for FI, who are not representative of the entire neonatal population, and therefore, the findings may not be generalizable to other populations. Furthermore, the study formulas differ not only in the protein equivalent component (free amino acids vs. extensively hydrolyzed protein), but also in other aspects (such as nutrient density, osmolarity, and minerals). These differences may potentially contribute to the differences in results. The study design does not permit us to elaborate on the different effects of each of these differences separately.

The large sample size is one of the greatest strengths of this study, the successful implementation of our study will provide robust evidence for formula alternatives in preterm infants with FI. This may further improve feeding intolerance and outcomes of preterm infants.

## Data Availability Statement

The original contributions presented in the study are included in the article/supplementary material, further inquiries can be directed to the corresponding author/s.

## Ethics Statement

Approval for this study has been granted by the Institutional Review Board of the Children's Hospital of Chongqing Medical University, China (File number: 68/2021). Written consent from both parents/guardians is mandatory to participate in this study.

## Author Contributions

QZ, QL, and NP developed the initial protocol and manuscript. X-HL provided consulting on statistical analysis. All authors read and approved the final manuscript.

## Conflict of Interest

The authors declare that the research was conducted in the absence of any commercial or financial relationships that could be construed as a potential conflict of interest.

## Publisher's Note

All claims expressed in this article are solely those of the authors and do not necessarily represent those of their affiliated organizations, or those of the publisher, the editors and the reviewers. Any product that may be evaluated in this article, or claim that may be made by its manufacturer, is not guaranteed or endorsed by the publisher.

## Abbreviations

AAF: Amino acid-based formula
BW: Birth weight
CRP: C-reactive protein
CRFs: case report forms
EHF: Extensively hydrolyzed formula
FI: Feeding intolerance
GA: Gestational age
HM: Human milk
HF: Hydrolyzed formula
NEC: necrotizing enterocolitis
PF: Preterm formula
PHF: Partially hydrolyzed formula.
